# Determinants of poor health-related quality of life among outpatients with rheumatoid arthritis in Jordan

**DOI:** 10.1371/journal.pone.0312557

**Published:** 2024-10-23

**Authors:** Anan S. Jarab, Walid Al-Qerem, Shrouq R. Abu Heshmeh, Karem H. Alzoubi, Yazid N. Al Hamarneh, Amal Akour

**Affiliations:** 1 College of Pharmacy, Al Ain University, Abu Dhabi, United Arab Emirates; 2 Faculty of Pharmacy, Department of Clinical Pharmacy, Jordan University of Science and Technology, Irbid, Jordan; 3 Faculty of Pharmacy, Department of Pharmacy, Al-Zaytoonah University of Jordan, Amman, Jordan; 4 Department of Pharmacy Practice and Pharmacotherapeutics, College of Pharmacy, University of Sharjah, Sharjah, UAE; 5 Faculty of Pharmacy, Jordan University of Science and Technology, Irbid, Jordan; 6 Faculty of Medicine and Dentistry, Department of Pharmacology, University of Alberta, Edmonton, Canada; 7 Department of Pharmacology and Therapeutics, College of Medicine and Health Sciences, Al Ain, United Arab Emirates; 8 Department of Biopharmaceutics and Clinical Pharmacy, School of Pharmacy, The University of Jordan, Amman, Jordan; National Research Centre, EGYPT

## Abstract

**Objective:**

The purpose of this study was to assess the health-related quality of life (HRQOL) and investigate the variables contributing to reduced HRQOL in patients with rheumatoid arthritis.

**Methods:**

The present cross-sectional study was conducted on patients diagnosed with rheumatoid arthritis at two teaching hospitals in Jordan using a convenience sampling technique. The participants were interviewed face-to-face during the scheduled appointment at the outpatient rheumatology clinic. The HRQOL was evaluated by the validated EuroQol-5 Dimension (EQ-5D) questionnaire, which included the EQ-5D utility index that evaluated HRQOL in terms of 5 domains, including mobility, self-care, usual activities, pain/discomfort, and anxiety/depression, and the EQ-5D visual analogue scale (EQ-5D_VAS_), which evaluated HRQOL on a vertical scale ranging from 0 (worst imaginable health) to 100 (best imaginable health). The validated short version of the 19-item Compliance Questionnaire for Rheumatology (CQR-5) was used to evaluate medication adherence. The Clinical Disease Activity Index (CDAI) was used to assess disease activity among the study participants. A stepwise quantile regression model (q = 0.5) was conducted to explore the factors associated with the EQ-5D_Utility Index_ and EQ-5D_VAS_ scores.

**Results:**

In total, 261 patients with RA participated in the study. The median (interquartile range) of the EQ-5D_Utility Index_ and EQ-VAS scores was 0.552 (0.006–0.726) and 0.506 (0.233–0.690), respectively. Regression analysis results demonstrated that medication non-adherence (regression coefficient (β) = -0.348, P<0.01), not performing regular physical activity (β = -0.209, P<0.01), and higher disease activity as measured by the CDAI score (β = -0.015, P<0.01) were significant predictors of a lower EQ-5D_Utility Index_ score_._ In addition, medication non-adherence (β = -0.199, P<0.01), not performing regular physical activity (β = -0.117, P<0.01), increased body mass index (BMI) (β = -0.009, P<0.01), and higher CDAI score (β = -0.009, P<0.01) were significant predictors of low EQ-5D_VAS_ score.

**Conclusions:**

Patients with RA experienced significantly impaired HRQOL. Medication non-adherence, not performing regular physical activity, increased body weight, and increased disease activity were identified as determinants of poor HRQOL among patients with RA in the present study. Treating physicians should encourage regular physical activity, maintaining a healthy body weight, and controlling disease activity to improve HRQOL in patients with RA.

## Introduction

Rheumatoid arthritis (RA) is a chronic autoimmune disease that develops when the body’s immune system, which typically aids in defending against illnesses and infections, attacks its tissues, resulting in pain, swelling, stiffness, and joint dysfunction [1]. Around 17.6 million individuals worldwide were estimated to have rheumatoid arthritis in 2020 [2]. In the Middle East and North Africa (MENA) region, age-standardized point prevalence for RA was 120.6 per 100,000 population in 2019, while the annual incidence rate was 5.9, representing increases of 28.3% and 25.2%, respectively, since 1990 [3]. Research on the prevalence of RA in Jordan is scarce, with only two reports available. One study conducted in the south of Jordan revealed an estimated prevalence of 0.36% [4], while another study in the north of Jordan reported a prevalence of 0.31% [5]. RA imposes a significant burden on the affected patients, as it can result in joint damage, functional disability, fatigue, depression, anxiety, and premature death, leading to significantly reduced work productivity and poor health-related quality of life (HRQOL) [6,7]. HRQOL is a comprehensive term that evaluates how an individual’s health status affects their overall quality of life, encompassing insights into both their physical and mental well-being [8]. The majority of RA patients have some degree of fatigue, which may be brought on by the chronic pain that is common in RA. Pain can cause fatigue, poor sleep, depression, and a low mood, all of which can lower HRQOL [9]. RA can also significantly affect other facets of human life, such as family life, social interactions, psychological health, and, hence, HRQOL. Additionally, patients with RA frequently find themselves unable to carry out daily tasks in both their personal and professional lives, and they should frequently change careers or retire early [10]. Based on the most recent data available on the worldwide burden of RA, the rate of age-standardized disability-adjusted life years (DALY) rose from 39.12 per 100,000 individuals in 1990 to 39.57 in 2019 [11]. In the MENA region, the burden of RA increased steadily between 1990 and 2019, underscoring the region’s ever-increasing burden of RA [3]. Aside from its burden on patients, RA treatment is costly and requires a variety of prescription drugs, including disease-modifying antirheumatic drugs (DMARDs), biologics, and painkillers, in addition to frequent follow-up appointments and monitoring. Most patients pay between $1,500 and $2,000 a year for DMARDs, depending on the medication. Biologics, on the other hand, can be very expensive, often costing between $1,300 and $3,000 a month. The cost is further increased by surgeries and rehabilitation, which includes physical and occupational therapy [12].

Previous research discovered that HRQOL was perceived as low among patients with RA, especially in the physical health domain. [13]. A systematic review and meta-analysis revealed that patients with RA in Asia generally exhibited low EuroQoL-5 Dimension (EQ-5D) utility scores, with an estimated average of 0.66 [14]. Another study conducted in Italy to compare HRQOL between four inflammatory rheumatic diseases reported that HRQOL was the worst among patients with RA [15]. Furthermore, reduced HRQOL scores were also reported among RA patients in several other studies [16–18].

In 2005, the World Health Organization (WHO) recognized the importance of evaluating and enhancing improved HRQOL [19], especially among those who suffer from severe diseases, given that patients who feel more comfortable in their lives will have the passion to fight the disease and improve their health [20]. In addition, a good quality of life will decrease patients’ concerns about discomfort, adverse effects, or other unpleasant aspects that could cause them to quit taking their medications or not take them as directed [20,21]. Therefore, it is essential to conduct research to determine the primary determinants of HRQOL in patients with RA, and it is even more crucial to develop efficacious strategies to improve their HRQOL afterwards.

Several factors, including disease activity, physical disability, psychosocial disturbances, body weight, comorbidities, extra-articular manifestations, delay in treatment onset, age, and marital status, were found to be significantly associated with HRQOL in patients with RA [22–25]. Currently, no previous studies have been conducted to investigate HRQOL and its associated factors in patients with RA in Jordan. With the aim of evaluating the level of HRQOL and identifying the factors that are associated with diminished HRQOL among RA patients in Jordan, the results of this study will contribute to the body of knowledge already available on HRQOL and its determinants in RA patients in the MENA region. Furthermore, the current research findings are expected to provide invaluable insights for developing successful RA management initiatives that are meant to improve the overall health and HRQOL of patients with RA. Additionally, the knowledge gained may be used to create interventions and programs specifically designed to address the challenges associated with RA, which would ultimately be advantageous to both patients and healthcare systems.

## Material and methods

### Study design and subjects

This cross-sectional study targeted patients diagnosed with RA at King Abdullah University Hospital (KAUH) and Prince Basma Hospital in Jordan between February and October 2021. Given the advantage of easy access to the participants, the patients were recruited using a convenience sampling technique. Patients older than 18 years and with an established diagnosis of RA according to the American College of Rheumatology/European Alliance of Associations for Rheumatology (ACR/EULAR) 2010 rheumatoid arthritis classification criteria [26] were eligible to participate. Other inclusion criteria included receiving a stable dose of at least one DMARD for four months or more and agreeing to participate in the study. The exclusion criteria included cognitive impairment and non-completion of the survey. The participants were interviewed face-to-face by the trained researcher (SRA) during the scheduled appointment at the outpatient rheumatology clinic. On average, it took ten to fifteen minutes to finish the self-reported questionnaire, and patients who had trouble reading the questions had the questions read aloud to them.

We utilized a tailored questionnaire to gather data on various sociodemographic variables. This included gender, marital status, education level, employment status, having health insurance, income, smoking, performing regular physical activity that involves engaging in any form of exercise, such as walking, cycling, or doing sports, for at least 30 minutes per day on most days of the week, and the presence of a family history of RA. In addition, we used medical records to retrieve disease- and medication-related information such as comorbidities, duration of RA, RA complications, and RA treatment details. This included the number, type, and frequency of administration of DMARD medications, alongside other medications used for RA treatment like nonsteroidal anti-inflammatory drugs (NSAIDs) or corticosteroids.

### Study instruments

#### The EuroQoL-5 Dimension

The validated EQ-5D instrument [27], which involves the EuroQol Group 5-Dimension 3-Level (EQ-5D-3L) and the EuroQol Visual Analogue Scale (EQ-VAS), was utilized to assess HRQOL. Previous research conducted among patients with RA confirmed that the EQ-5D tool is valid, easy to use, responsive to change, and sufficiently reliable for group comparisons [28]. Additionally, it was reported that, with the exception of the Health Assessment Questionnaire, its reliability is equal to or better than that of all other instruments [28]. The validity and reliability of the Arabic version of the EQ-5D instrument have been established in previous studies conducted in Saudi Arabia [29] and Jordan. [30], where the Cronbach’s α values were 0.72 and 0.75, respectively, indicating an acceptable level of internal consistency of the Arabic version of the questionnaire [29,30]. The EQ-5D-3L consists of five domains, including mobility “I have no problems walking”, “I have some problems walking”, “I am attached to the bed and I do not walk”; self-care “I have no problems with self-care”, “I have some problems washing and wearing clothes myself”, “I cannot wash or dress clothes myself”; usual activities “I have no problems performing my usual activities”, “I have some problems performing my usual activities”, “I cannot perform my usual activities”; pain/discomfort “I have no pain or discomfort”, “I have mild pain or discomfort”, “I have severe and excessive pain”, and anxiety/depression “I am not worried or frustrated (depressed)”, “I am moderately depressed or anxious”, “I am very depressed and anxious”. The patients were asked to indicate if they had no problem (coded 1), some problem (coded 2), or an extreme problem (coded 3) in each part. The resultant 5-digit code was subsequently transformed into an index value that ranges from 0 to 1 according to the value set of the United Kingdom, with higher values indicating better HRQOL. These value sets are generated using a variety of methods, such as the VAS and the Time-Trade-Off (TTO) method. In the TTO method, participants are asked to imagine themselves living for ten years in a certain state of suboptimal health and then indicate how long they would be willing to give up in order to live in full health. When using the VAS technique, participants are asked to place a health state on a vertical, graduated scale that goes from the best possible health to the worst possible health [31]. Hence, the index value based on the TTO approach is represented by the EQ-5D_utility index_, and the index value based on the VAS technique is represented by the EQ-5D_VAS_.

The EQ-VAS recorded participants’ self-assessed health on a vertical scale ranging from 0 to 100, where 0 means “the worst health you can imagine” and 100 means “the best health you can imagine.” Participants were instructed to mark a point on this scale to indicate their current health status.

#### The 5-item Compliance Questionnaire for Rheumatology

The CQR-5 is a validated short version of the 19-item Compliance Questionnaire for Rheumatology (CQR-19), which is the only self-report adherence measure specifically created and validated for rheumatic diseases. The CQR-5 has been approved for validation and reliability in detecting non-adherence to antirheumatic medications [32]. It has been used to assess medication non-adherence among patients with RA in previous studies [33,34]. The Arabic version of the CQR-5 has demonstrated validity and reliability to assess medication adherence in patients with RA in Saudi Arabia [35]. The CQR-5 evaluates medication adherence on a 4-point Likert scale ranging from; “definitely don’t agree” (scored 1) to “definitely agree” (scored 4), with lower scores indicating lower levels of adherence. Based on two distinct formulas for classifying adherence, participants were categorized into either high or low adherent groups. The formula for high adherence is D1 = -33.304 + (2.801 * Q1) + (5.008 * Q2) + (6.471 * Q3) + (1.215 * Q4) + (3.252 * Q5), while the formula for low adherence is D0 = -27.611 + (4.407 * Q1) + (0.939 * Q2) + (6.101 * Q3) + (2.366 * Q4) + (2.531 * Q5), the participants were classified as either low adherents or high adherents. If D0 was greater than D1, the participant was classified as a low adherent. On the other hand, the participant was categorized as highly adherent if D1 was greater than D0 [32]. The variable Q in each formula represents a “question”.

#### Beliefs about Medication Questionnaire

The validated Arabic version of the specific part of the two distinct five-item Beliefs about Medication Questionnaire (BMQ), with a Cronbach’s alpha of 0.89–0.93, demonstrates the reliability of the instrument. The tool evaluated medication beliefs in terms of necessity and concerns on a five-point Likert scale ranging from “strongly disagree” (score 1) to “strongly agree” (score 5) [36]. Scores for individual items on each scale were summed, resulting in a scale score ranging from 5 to 25, where higher scores indicated stronger beliefs in the respective domains [37].

#### The Clinical Disease Activity Index

The Clinical Disease Activity Index (CDAI) is a validated and reliable tool used to assess disease activity in RA patients [38]. It evaluates the number of swollen joints, tender joints, the patient’s self-assessed global disease activity (on a scale of 1–10), and the evaluator’s global disease activity assessment (also on a scale of 1–10). The physician utilized the CDAI to classify the patients as having low (scores from 3 to 10), moderate (more than 10 and up to 22), or high (more than 22) disease activity [39].

#### Sample size calculation

The sample size calculation of the present study was based on a sound statistical approach that was adopted in several previous studies [40,41]. The Green equation 50 + 8P was applied to compute the minimal sample size, where P represents the number of predictors [42]. The model included 25 predictors; therefore, the minimal required sample size was 250.

#### Data analysis

Data was analyzed using the Statistical Package for the Social Sciences (SPSS version 27, IBM, Chicago, Illinois, USA) and the quantile regression package in R version 5.79. According to the Q-Q plots and Kolmogorov–Smirnov test, normally distributed continuous variables were presented as mean (standard deviation [SD]), and non-normally distributed variables were presented as median (interquartile range [IQR]). The categorical variables were presented as frequencies and percentages. The Q-Q plots and the Kolmogorov-Smirnov test revealed a non-normal distribution (p<0.001) of the EQ-5D_Utility Index_ and EQ-5D_VAS_ scores, and therefore, nonparametric analysis and quantile regression were conducted. The dependent variables were both the EQ-5D_Utility Index_ and EQ-5D_VAS_ scores. The independent variables were the sociodemographic data, disease-related characteristics, medication-related characteristics, medication adherence level, disease activity level, and the BMQ scores. The Mann-Whitney U-test was conducted to identify the dichotomous independent variables associated with EQ-5D_Utility Index_ and EQ-5D_VAS_ scores. The Kruskal-Wallis test was used to identify the association between multinomial independent variables and EQ-5D_Utility Index_ and EQ-5D_VAS_ scores. Spearman’s rank correlation was used to identify the continuous independent variables associated with the EQ-5D_Utility Index_ and EQ-5D_VAS_ scores. After the inclusion of variables with P<0.05 from the bivariate analysis, a stepwise quantile regression model (q = 0.5) was conducted after adjustment for age, gender, disease duration, and other comorbid diseases. Model estimation was performed using the Simplex algorithm developed by Barrodale and Roberts [43]. The variance-covariance matrix of the parameter estimates was produced using the Bofinger bandwidth method [43]. The regression coefficients produced were interpreted as the impact of a one-unit change in the predictor on the adherence score (median difference) while holding other predictors fixed. The thresholds for α and p-values in both bivariate and multivariate analyses were set at 0.05.

#### Ethical approval statement

The Institutional Review Board (IRB) of KAUH granted ethical permission for the study (Ref. # 58/132/2020). Participants were assured that their participation in the study was entirely voluntary, and they had the right to withdraw at any time. They were also assured that their medical care at the hospital would not be impacted by their participation. The researcher emphasized that all data collected would be used solely for research purposes and would be stored confidentially in the principal investigator’s office. All individuals who agreed to participate provided written, informed consent.

## Results

Out of 313 patients, 261 agreed to participate (response rate = 83.4%), of whom 86.6% were female. The age range of the study participants was 19 to 83 years, with a mean (SD) age of 48.7 (12.57) years. The mean (SD) body mass index (BMI) was 30.04 (6.52). Most of the study patients were married (77%), had a low level of education (63.6%), were not employed (72.4%), or retired (10.7%), had a low income (<700 USD) (64%), and had health insurance (78.5%). On the other hand, only 19.9% of the patients were smokers, and 21.5% exercised regularly, respectively. The socio-demographic characteristics of the study patients are presented in Table 1.

**Table 1:** Socio-demographic characteristics of the study participants (n = 261).

Characteristics		N (%)
Gender	Male	35 (13.4)
	Female	226 (86.6)
Marital status	Not married	60 (22)
	Married	201 (77)
Education^a^	Less than Diploma degree (low)	166 (63.6)
	Diploma degree or higher (high)	95 (36.4)
Employment	Employed	44 (16.9)
	Not employed	189 (72.4)
	Retired	28 (10.7)
Having health insurance	Yes	205 (78.5)
	No	56 (21.5)
Income	Less than 700 USD	167 (64.0)
	700 USD– 1400 USD	82 (31.4)
	More than 1400 USD	12 (4.6)
Smoking	Nonsmoker	201 (77)
	Current smoker	52 (19.9)
	Ex-smoker	8 (3.1)
Performing regular physical activity ^b^	Yes	56 (21.5)
	No	205 (78.5)
Having a family history of RA	Yes	70 (26.8)
	No	191 (73.2)

^a^ Education: Less than a diploma degree includes illiteracy, primary school, and secondary school. A higher than diploma degree includes bachelor, master, or PhD degrees.

^b^ Regular physical activity was engaging in any form of exercise, such as walking, cycling, or sports, for at least 30 minutes per day on most days of the week. Participants who self-reported meeting this criterion were classified as performing regular physical activity; those who did not were classified as not.

As shown in Table 2, the predominant comorbidity identified among the participants in the study was hypertension, affecting 29.5% of the patients, with diabetes following closely at 20.3%. The study participants displayed a moderate level of RA activity, manifested by a median CDAI score of 19. Notably, 37.5% of these patients had a highly active form of the disease. The median duration since the diagnosis of RA was 10 years (4–16.5). The shortest duration of the disease was 6 months, while the longest duration was 40 years. Additionally, RA complications affected a substantial majority of the study population, with 93.1% experiencing various complications. Among these complications, peripheral neuropathy was the most prevalent, affecting 77.4% of the patients. Eye problems were present in approximately half of the participants (46.7%), osteoporosis affected 37.5%, and joint deformity was observed in 21.1%. More than half of the participants were treated with a single DMARD (51.7%), which could be either conventional or biologic, while 44.1% of the participants who were on DMARD treatment had their medication administered on a weekly basis. The majority of the patients were on methotrexate therapy (67.8%), with a median dose of 15 mg/week (12.5–20). Of those receiving methotrexate, 97.2% were taking it orally. Around 36.8% received biologic DMARDs, while none of the participants were prescribed targeted synthetic DMARDs. Furthermore, 75.1% of the patients used NSAIDs, or corticosteroids, for pain relief. According to the CQR-5 assessment, it was determined that 43.3% of the study participants showed poor medication adherence.

**Table 2:** Disease and medication characteristics of the study participants (n = 261).

Variable			N (%)
RA therapy	Conventional DMARDs	Methotrexate	177 (67.8)
		Sulfasalazine	73 (28)
		Hydroxychloroquine	32 (12.3)
		Azathioprine	14 (5.4)
	Biologic DMARDs		96 (36.8)
	Analgesics		196 (75.1)
Number of DMARDs	1		135 (51.7)
	2		96 (36.8)
	3		27 (10.3)
	4		3 (1.2)
Frequency of DMARD administration	Once per month		1 (0.4)
	Once per two weeks		7 (2.7)
	Once weekly		115 (44.1)
	Once daily		48 (18.4)
	Twice daily		90 (34.5)
Presence of comorbid diseases	Yes		177 (67.8)
	No		84 (32.2)
Type of comorbidities	Hypertension		77 (29.5)
	Diabetes mellitus		53 (20.3)
	Chronic respiratory disease		24 (9.2)
	Hypothyroidism		20 (7.7)
Presence of RA complications	Yes		243 (93.1)
	No		18 (6.9)
Type of complications	Joint deformity		55 (21.1)
	Arthroplasty		36 (13.8)
	Peripheral neuropathy		202 (77.4)
	Osteoporosis		98 (37.5)
	Eye problems		122 (46.7)
	Cardiovascular disease		13 (5.0)
Activity of the disease	Low		53 (20.3)
	Moderate		88 (33.7)
	High		98 (37.5)
	Missing		22 (8.5)
**Variable**			**Median (interquartile range)**
Duration since RA diagnosis (years)			10 (4.0–16.5)
Number of comorbidities other than RA			2.0 (1.0–3.0)
Number of RA medications			2.0 (2.0–3.0)
Number of total medications			6.0 (4.0–8.0)
Duration of medications intake			8.0 (2.0–14.0)
ESR (mm/hour)			44.0 (30.0–65.0)
CDAI score			19.0 (11.0–26.0)
**Medication adherence** ^**a**^			**N (%)**
High			148 (56.7)
Low			113 (43.3)

RA: Rheumatoid arthritis, CDAI: Clinical disease activity index, DMARD: Disease-modifying anti-rheumatic drug, ESR: Erythrocyte sedimentation rate.

^a^ Participants were classified as having a high or low medication adherence level based on the Compliance Questionnaire for Rheumatology 5-item (CQR-5).

**Fig 1:**
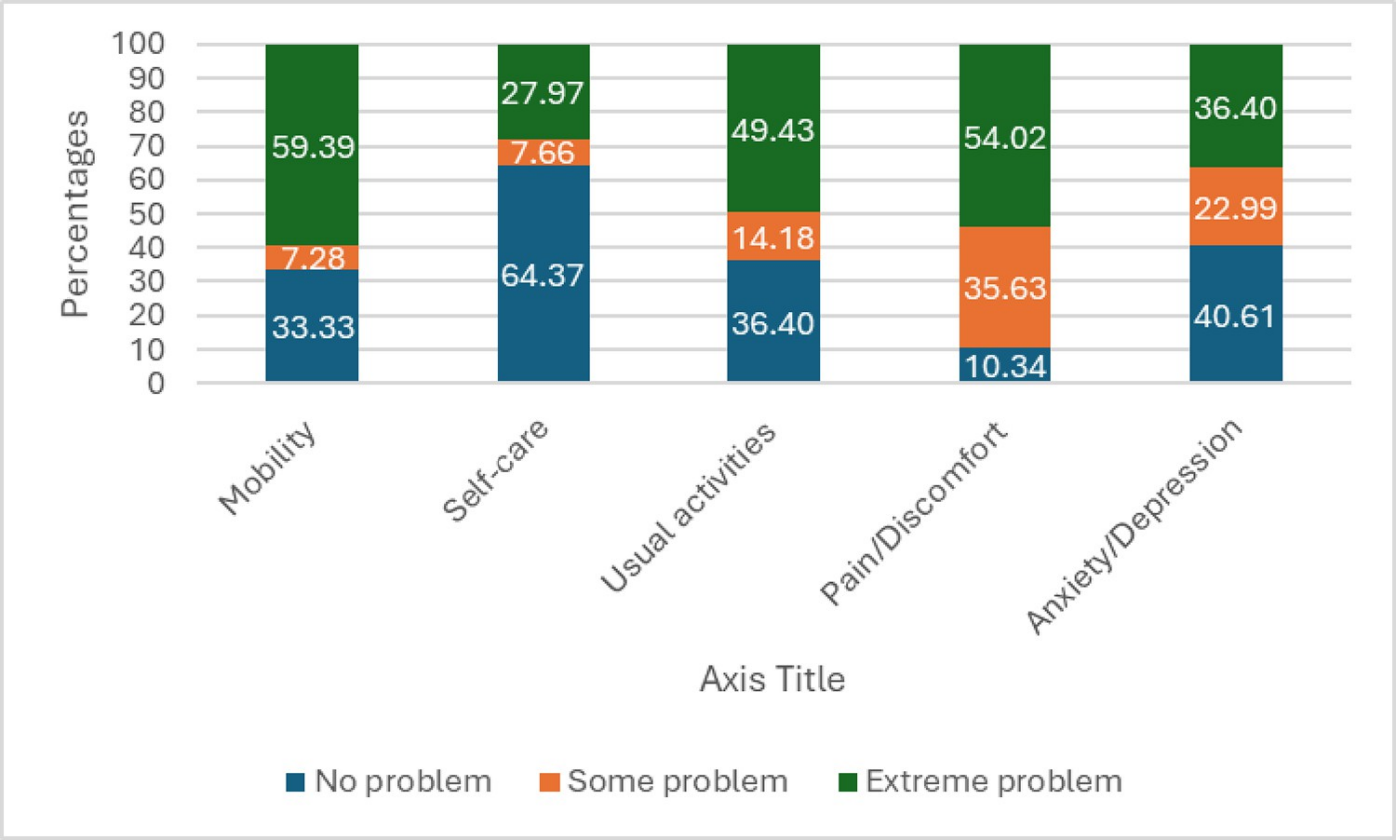
Distribution of the study participants according to the EQ-5D.

Regarding HRQOL assessment, the median EQ-5D_Utility Index_ was 0.552 (-0.006–0.726), while the median EQ-5D_VAS_ score was 0.506 (0.233–0.690). As shown in Fig 1, a significant portion of the participants reported experiencing extreme problems with walking (59.4%), pain or discomfort (54.0%), and performing their usual activities (49.4%). On the other side, most participants indicated they had no difficulties with self-care (64.4%) and reported no signs of anxiety or depression (40.6%). The median EQ VAS score, which stood at 40 (20–60), pointed to a moderate level of health status within the study’s patient population.

According to the BMQ, the participants demonstrated high beliefs regarding the necessity of their medications, with a median score of 20 (5–25). Regarding the concern score, the participants exhibited low beliefs, with a median score of 14 (5–25).

The bivariate analysis revealed that the EQ-5D_Utility Index_ score was significantly associated with each of the following factors: education level, income, physical activity, family history for RA, the presence of RA complications, eye problems, peripheral neuropathy, joint deformity, cardiovascular complications, arthroplasty, diabetes, receiving corticosteroids and/or NSAIDs, medication non-adherence, disease activity, ESR level, and the number of complications. On the other hand, the EQ-5D_VAS_ score was significantly associated with each of the following factors: education level, income, physical activity, the presence of RA complications, eye problems, peripheral neuropathy, joint deformity, cardiovascular complications, arthroplasty, hypertension, receiving corticosteroids and/or NSAIDs, medication non-adherence, disease activity, ESR level, and the number of complications. More details on the bivariate analysis results are presented in S1 Table.

Results of the stepwise quantile regression analysis are presented in Table 3. The CDAI score was significantly associated with the EQ-5D utility index (β = -0.015, 95%CI = -0.020 - -0.010, P<0.001) and the EQ-5D VAS (β = -0.009, 95%CI = -0.012 - -0.006, P<0.001), indicating that one unit increase in the CDAI score was associated with a 0.015 decrease in the EQ-5D utility index and a 0.009 decrease in the EQ-5D VAS scores. In addition, each unit increase in BMI was associated with a 0.009 decrease in the EQ-5D VAS score (β = -0.009, 95%CI = -0.014 - -0.003, P<0.013). When compared with the high adherence group, being in the low adherence group was associated with a decrease in the EQ-5D utility index (β = -0.348, 95%CI = -0.459 - -0.238, P<0.001) and the EQ-5D VAS (β = -0.199, 95%CI = -0.266 - -0.131, P<0.001) by 0.348 and 0.199, respectively. Lastly, not performing regular physical activity decreased the EQ-5D utility index by 0.209 (β = -0.209, 95%CI = -0.329 - -0.078, P<0.025) and the EQ-5D VAS by 0.117 (β = -0.199, 95%CI = -0.266 - -0.131, P<0.001). The adjusted variables, including age, gender, disease duration, and other comorbid diseases, were not significantly associated with the EQ-5D_Utility Index_ or the EQ-5D_VAS_ score. The pseudo-R^2^ values for the EQ-5D_Utility Index_ and EQ-5D_VAS_ were 0.266 and 0.285, respectively, demonstrating a very good model fit. Due to the large number of investigated independent variables, stepwise quantile regression analysis was conducted in the present study. This type of regression displays only the significant variables in the final step. Therefore, the regression analysis results (Table 3) did not include the independent variables that had a non-significant association with the dependent variables in this study.

**Table 3:** Multivariate quantile regression of the factors associated with HRQOL*.

	EQ-5D_Utility Index_				EQ-5D_VAS_			
	β ^a^	P	95% CI		β ^a^	P	95% CI	
	
			lower	upper			lower	upper
CDAI score	-0.015	<0.001	-0.020	-0.010	-0.009	<0.001	-0.012	-0.006
Body mass index score	-0.010	0.054	-0.019	-0.001	-0.009	0.013	-0.014	-0.003
Low vs. high adherence**	-0.348	<0.001	-0.459	-0.238	-0.199	<0.001	-0.266	-0.131
Not performing vs. performing regular physical activity***	-0.209	0.025	-0.329	-0.078	-0.117	0.018	-0.196	-0.038

CDAI: Clinical disease activity index. ^a^ Quantile regression coefficient.

*The independent variables included in the EQ-5DUtility Index regression model were education level, income, physical activity, family history for RA, the presence of RA complications, eye problems, peripheral neuropathy, joint deformity, cardiovascular complications, arthroplasty, diabetes, receiving corticosteroids and/or NSAIDs, medication non-adherence, disease activity, ESR level, and the number of complications. The independent variables included in the EQ-5DVAS regression model were education level, income, physical activity, the presence of RA complications, eye problems, peripheral neuropathy, joint deformity, cardiovascular complications, arthroplasty, hypertension, receiving corticosteroids and/or NSAIDs, medication non-adherence, disease activity, ESR level, and the number of complications.

** Based on two distinct formulas for classifying adherence, participants were categorized into either high or low adherent groups. The formula for high adherence is D1 = -33.304 + (2.801 * Q1) + (5.008 * Q2) + (6.471 * Q3) + (1.215 * Q4) + (3.252 * Q5), while the formula for low adherence is D0 = -27.611 + (4.407 * Q1) + (0.939 * Q2) + (6.101 * Q3) + (2.366 * Q4) + (2.531 * Q5), the participants were classified as either low adherents or high adherents. If D0 was greater than D1, the participant was classified as a low adherent. On the other hand, the participant was categorized as highly adherent if D1 was greater than D0. [32]. The variable Q in each formula represents a “question”.

*** Regular physical activity included engaging in any form of exercise, such as walking, cycling, or sports, for at least 30 minutes per day on most days of the week.

## Discussion

The present research findings showed a diminished HRQOL among patients with RA. Several factors have been identified as significant determinants of this decline, including higher body weight, increased disease activity, limited physical activity, and poor adherence to the treatment regimen.

Comparable with the current study EQ-5D value, earlier studies reported low HRQOL with a mean EQ-5D of 0.6 and 0.65 among patients with RA in Korea and Thailand, respectively. [44,45]. Other studies reported higher EQ-5D scores among patients with RA [17,22,46]. Poor HRQOL was also reported in studies using instruments other than the EQ-5D. According to a Saudi study that used the World Health Organization Quality of Life (WHOQOL-BRIEF) tool, low overall HRQOL was reported in patients with RA [13]. Another study conducted in Poland reported moderate HRQOL among patients with RA, as estimated by the WHOQOL-BREF [47]. An Italian study, using the 36-item short instrument, demonstrated that HRQOL scores were the worst in the RA group compared to groups with other rheumatic diseases [15]. Furthermore, a study conducted in China using multiple instruments, including the WHOQOL-BREIF, the SF-36, and the Quality of Life Instruments for Chronic Diseases-RA (QLICD-RA), showed lower HRQOL in RA patients when compared to healthy individuals [23]. When considering other medical conditions as well as normative datasets from populations in the UK and the USA, a systematic review and meta-analysis reported that patients with RA have a significantly lower HRQOL, measured by the SF-36 [48].

Controversial findings have been reported regarding the relationship between BMI and HRQOL in patients with RA. A large Korean investigation, which enrolled about two thousand women with RA, found that the patients who had the highest BMI reported the lowest EQ-5D score [17]. In contrast, a Chinese study reported a beneficial effect of high BMI on RA patients’ HRQOL [23]. A higher BMI was associated with lower HRQOL in the present study patients. A higher BMI has been correlated with increased levels of various inflammatory mediators, which contribute to heightened disease activity [49]. An earlier study reported a significant association between increased RA severity and poor HRQOL in RA patients [50], which justifies the significant association between increased BMI and poor HRQOL in the present study.

In line with earlier research findings [18,22,51–53], our study showed that participants who had higher RA activity estimated by the CDAI score reported lower HRQOL than those with less active disease. Even though RA is a progressive disease, uncontrolled RA can cause irreversible joint damage, leading to reduced functional capacity and poor HRQOL [54]. An earlier study reported that most RA patients who had high disease activity were depressed and that functional disability increased considerably with disease activity [16]. In addition, another Egyptian study reported a positive correlation between disease activity and both anxiety and depression among patients with RA [51]. Therefore, controlling RA activity may significantly improve HRQOL and lower the chance of developing irreparable joint deterioration and functional impairment, in addition to improving mental health in patients with RA.

Low physical activity was negatively correlated with HRQOL in the current research. Similar findings were found in a Korean investigation, which showed that patients with RA who did not regularly exercise had significantly worse EQ-5D scores [17]. Earlier studies conducted in the USA also reported impaired HRQOL among arthritis patients with low physical activity [55,56]. Regular exercise has been associated with specific health benefits for patients with RA. It can help improve joint flexibility and strength, reduce pain, and stimulate weight loss, which is also good for RA because weight gain can worsen symptoms by putting more pressure on already inflamed joints [57] and increasing the risk of cardiovascular diseases [58].

In the present investigation, low adherence to the RA treatment regimen was linked to diminished HRQOL. Non-adherence to RA medications has been associated with increased disease activity and joint damage [59], which would worsen patients quality of life if not optimized. Several studies have reported the negative impact of non-adherence on HRQOL in RA patients [59,60]. In a study of biologic DMARD-using RA patients, it was discovered that individuals who did not comply with their treatment plan as directed had higher levels of physical impairment and a lower HRQOL than those who did [61]. Therefore, appropriate interventional strategies should be taken to improve medication adherence among RA patients in order to optimize therapeutic outcomes, prevent negative impacts on disease activity and joint damage, and ultimately improve HRQOL.

### Strengths and limitations

The present study findings shed light on the necessity of enhancing medication adherence and encouraging physical activity, particularly among patients with increased body weight and more severe RA, as a preliminary step for the development of future interventions aimed at enhancing HRQOL in patients with RA. In addition, the study utilized a broad approach to analyze different spheres of influence, as living with RA is complex and there are diverse factors that possibly affect HRQOL. The use of validated and standardized instruments to assess HRQOL and most of its potential predictors represented further strength of the study. Furthermore, the researchers adopted several strategies to ensure the high quality of the collected data. The researcher who collected the data received training sessions about communication skills, the university’s ethical guidelines, data collection procedures, and survey delivery strategy. The researcher was assured of avoiding unintentional leading or suggestive behavior and advising the patient to read and answer the questions independently, unless support in terms of reading the questions or the patient who was unable to do so independently requested typing the answers. In addition, two other different researchers entered the data independently to minimize data entry errors before data analysis, and any discrepancies between entries were resolved through reconciliation. The researchers also processed the data to identify and correct any errors, outliers, or missing values to ensure the high quality of the dataset before running statistical analysis.

However, the study has some limitations that must be acknowledged. The cross-sectional design of the study does not allow the assessment of temporal or causal relationships between the predictors and outcomes. The use of a convenience sampling technique, recruiting patients exclusively from two hospitals in Jordan, and the lack of a homogeneous sample due to the higher proportion of female participants are all factors that potentially represent selection bias and affect the generalizability of the study findings. The self-reported instrument may overestimate and not accurately reflect patients’ true behaviors, leading to social-desirability bias. However, self-reported measures are considered important in clinical research evaluating HRQOL [62]. Furthermore, the EQ-5D instrument has its own limitations, even though it has been used in several prior studies to evaluate HRQOL in a variety of diseases. It was reported that not all of the components identified as shaping HRQOL are fully captured by the measure. It might also be ineffective in evaluating disease symptoms that fluctuate. Furthermore, the instrument demonstrated a lack of sensitivity in the assessment of HRQOL in several diseases, including those deemed mild or asymptomatic [63]. Future research that involves recruiting a larger number of patients with RA is recommended to draw more generalizable and robust conclusions from the study findings. Furthermore, it is deemed necessary to implement randomized controlled trial studies to evaluate the impact of the interventions that utilize the determinants of HROL identified in the present study on health outcomes among patients with RA.

## Conclusion

This study clearly demonstrated poor HRQOL among outpatient individuals with RA. To improve HRQOL among RA patients, forthcoming disease management initiatives should prioritize tailored strategies that enhance medication adherence and promote regular physical activity, with specific attention to overweight and obese patients and those grappling with high disease activity. Furthermore, achieving and sustaining disease control, particularly in individuals with highly active disease and multiple complications, is of great importance for the improvement of HRQOL, emphasizing the necessity for personalized interventions and regular monitoring of these patients.

## Supporting information

S1 Checklist (DOCX)

S2 Checklist STROBE statement—checklist of items that should be included in reports of *cross-sectional studies*. (DOCX)

S1 Table (DOCX)

S1 Data (SAV)
