# Supplementary material for: Determinants of poor health-related quality of life among outpatients with rheumatoid arthritis in Jordan
Source: PLoS One. 2024 Oct 23;19(10):e0312557. doi: 10.1371/journal.pone.0312557 (PMC11498696; doi:10.1371/journal.pone.0312557)
Supplement: S1 Table — (DOCX) [file pone.0312557.s003.docx]

**Supplementary data:**

**Table S1:** Results of Univariate analysis of factors associated with HRQOL.

| **Variable** | | | | | | **Median EQ-5D_Utility Index_ (25%-75%)** | | **P-value** | **Median EQ-5D_VAS_ (25%-75%)** | | **P-value** |
| --- | --- | --- | --- | --- | --- | --- | --- | --- | --- | --- | --- |
| Gender | | Male | | | | 0.656 (0.055-0.779) | | 0.154 | 0.627 (0.264-0.730) | | 0.155 |
|  |  | Female | | | | 0.516 (-0.010-0.725) | |  | 0.503 (0.233-0.690) | |  |
| Marital status | | Married | | | | 0.585 (0.0035-0.725) | | 0.902 | 0.565 (0.264-0.69) | | 0.795 |
|  |  | Single** | | | | 0.516 (-0.0218-0.7518) | |  | 0.503 (0.1908-0.722) | |  |
| Health insurance | | No | | | | 0.516 (-0.0763-0.7265) | | 0.849 | 0.503 (0.203-0.690) | | 0.821 |
|  |  | Yes | | | | 0.585 (0.026-0.726) | |  | 0.565 (0.264-0.690) | |  |
| Living conditions | | Alone | | | | 0.082 (-0.2145-0.6215) | | 0.135 | 0.264 (0.128-0.5965) | | 0.163 |
|  |  | Live with family members | | | | 0.585 (0.0163-0.727) | |  | 0.5495 (0.2445-0.69) | |  |
| Education level*** | | Low | | | | 0.2775 (-0.077-0.691) | | **0.001*** | 0.3965 (0.209-0.667) | | **0.001*** |
|  |  | High | | | | 0.62 (0.88-0.727) | |  | 0.596 (0.294-0.698) | |  |
| Occupation | | Employed | | | | 0.534 (0.082-0.727) | | 0.529 | 0.52 (0.264-0.690) | | 0.542 |
|  |  | Un-employed | | | | 0.585 (-0.022-0.725) | |  | 0.506 (0.233-0.69) | |  |
| Smoking | | No | | | | 0.516 (-0.016-0.725) | | 0.097 | 0.503 (0.233-0.690) | | 0.096 |
|  |  | Yes | | | | 0.6035 (0.101-0.796) | |  | 0.585 (0.307-0.759) | |  |
| Healthy diet | | No | | | | 0.516 (-0.010-0.727) | | 0.946 | 0.503 (0.233-0.69) | | 0.665 |
|  |  | Yes | | | | 0.587 (-0.003-0.725) | |  | 0.566 (0.264-0.69) | |  |
| Income**** | | Low | | | | 0.195 (-0.077-0.689) | | **<0.0001*** | 0.357 (0.201-0.659) | | **<0.0001*** |
|  |  | High | | | | 0.673 (0.264-0.796) | |  | 0.648 (0.442-0.754) | |  |
| Performing regular physical activity | | No | | | | 0.362 (-0.041-0.691) | | **<0.0001*** | 0.459 (0.213-0.667) | | **<0.0001*** |
|  |  | Yes | | | | 0.691 (0.3015-0.796) | |  | 0.659 (0.470-0.761) | |  |
| Family history | | No | | | | 0.516 (-0.016-0.691) | | **0.042*** | 0.503 (0.233-0.667) | | 0.089 |
|  |  | Yes | | | | 0.620 (0.0978-0.7353) | |  | 0.596 (0.288-0.706) | |  |
| **RA complications** | | | | | | | | | | | |
| **Presence of any complications of RA** | | | | No | | 0.725 (0.3833-0.848) | | **0.006*** | 0.698 (0.492-0.782) | | **0.003*** |
|  |  |  |  | Yes | | 0.516 (-0.016-0.725) | |  | 0.503 (0.233-0.69) | |  |
| Eye problems | | | | No | | 0.62 (0.088-0.727) | | **0.002*** | 0.596 (0.294-0.698) | | **0.002*** |
|  |  |  |  | Yes | | 0.274 (-0.077-0.689) | |  | 0.3895 (0.182-0.659) | |  |
| Peripheral neuropathy | | | | No | | 0.689 (0.101-0.796) | | **0.003*** | 0.659 (0.301-0.761) | | **0.003*** |
|  |  |  |  | Yes | | 0.516 (-0.041-0.691) | |  | 0.503 (0.233-0.667) | |  |
| Arthroplasty | | | | No | | 0.587 (0.0415-0.727) | | **0.009*** | 0.566 (0.264-0.694) | | **0.002*** |
|  |  |  |  | Yes | | 0.1415 (-0.181-0.62) | |  | 0.310 (0.109-0.591) | |  |
| Joint deformity | | | | No | | 0.604 (0.055-0.727) | | **0.001*** | 0.585 (0.264-0.698) | | **<0.0001*** |
|  |  |  |  | Yes | | 0.128 (-0.157-0.62) | |  | 0.272 (0.140-0.596) | |  |
| Osteoporosis | | | | No | | 0.585 (0.015-0.725) | | 0.559 | 0.566 (0.264-0.69) | | 0.377 |
|  |  |  |  | Yes | | 0.516 (-0.022-0.727) | |  | 0.503 (0.231-0.69) | |  |
| CVD | | | | No | | 0.586 (0.002-0.727) | | **0.036*** | 0.566 (0.245-0.690) | | **0.035*** |
|  |  |  |  | Yes | | 0.101 (-0.146-0.570) | |  | 0.307 (0.183-0.550) | |  |
| **Presence of chronic diseases other than RA** | | | | No | | 0.586 (-0.016-0.725) | | 0.901 | 0.574 (0.241-0.698) | | 0.614 |
|  |  |  |  | Yes | | 0.516 (0.018-0.727) | |  | 0.503 (0.233-0.69) | |  |
| Hypertension | | | | No | | 0.620 (0.022-0.727) | | 0.052 | 0.585 (0.264-0.698) | | **0.047*** |
|  |  |  |  | Yes | | 0.264 (-0.077-0.689) | |  | 0.402 (0.233-0.659) | |  |
| Diabetes mellitus | | | | No | | 0.587 (0.016-0.727) | | **0.047*** | 0.574 (0.264-0.698) | | **0.047*** |
|  |  |  |  | Yes | | 0.264 (-0.077-0.620) | |  | 0.388 (0.214-0.596) | |  |
| Hypothyroidism | | | | No | | 0.585 (0.009-0.725) | | 0.918 | 0.534 (0.251-0.69) | | 0.964 |
|  |  |  |  | Yes | | 0.356 (-0.065-0.779) | |  | 0.446 (0.201-0.743) | |  |
| Chronic respiratory disease | | | | No | | 0.585 (-0.006-0.727) | | 0.273 | 0.565 (0.233-0.690) | | 0.296 |
|  |  |  |  | Yes | | 0.356 (0.002-0.622) | |  | 0.446 (0.217-0.597) | |  |
| Herniated disc | | | | No | | 0.534 (-0.016-0.727) | | 0.614 | 0.506 (0.233-0.690) | | 0.755 |
|  |  |  |  | Yes | | 0.585 (0.028-0.656) | |  | 0.574 (0.294-0.627) | |  |
| **RA medications** | | | | | | | | | | | |
| Methotrexate | | | | | No | 0.516 (0.042-0.727) | 0.811 | | | 0.503 (0.272-0.698) | 0.615 |
|  |  |  |  |  | Yes | 0.585 (-0.016-0.726) |  |  |  | 0.566 (0.233-0.690) |  |
| hyroxychloroquine | | | | | No | 0.552 (-0.006-0.726) | 0.651 | | | 0.506 (0.233-0.690) | 0.511 |
|  |  |  |  |  | Yes | 0.568 (-0.017-0.727) |  |  |  | 0.550 (0.264-0.698) |  |
| Sulfasalazine | | | | | No | 0.516 (-0.016-0.727) | 0.755 | | | 0.503 (0.233-0.690) | 0.763 |
|  |  |  |  |  | Yes | 0.587 (0.042-0.725) |  |  |  | 0.566 (0.264-0.690) |  |
| Azathioprine | | | | | No | 0.516 (0.015-0.725) | 0.576 | | | 0.506 (0.233-0.690) | 0.500 |
|  |  |  |  |  | Yes | 0.691 (-0.022-0.744) |  |  |  | 0.659 (0.211-0.709) |  |
| Biologic DMARDs | | | | | No | 0.516 (-0.016-0.727) | 0.985 | | | 0.506 (0.236-0.690) | 0.795 |
|  |  |  |  |  | Yes | 0.586 (0.002-0.725) |  |  |  | 0.550 (0.233-0.690) |  |
| Corticosteroids/ NSAIDs | | | | | No | 0.656 (0.221-0.796) | **0.002*** | | | 0.627 (0.361-0.761) | **0.001*** |
|  |  |  |  |  | Yes | 0.465 (-0.038-0.691) |  |  |  | 0.481 (0.227-0.667) |  |
| **Number of DMARDs** | Single | | | | No | 0.534 (-0.004-0.725) | 0.556 | | | 0.506 (0.231-0.690) | 0.382 |
|  |  |  |  |  | Yes | 0.585 (-0.016-0.727) |  |  |  | 0.565 (0.264-0.698) |  |
|  | Double | | | | No | 0.516 (-0.016-0.725) | 0.112 | | | 0.503 (0.233-0.690) | 0.232 |
|  |  |  |  |  | Yes | 0.620 (0.075-0.727) |  |  |  | 0.581 (0.264-0.692) |  |
|  | Triple or more | | | | No | 0.569 (0.023-0.727) | 0.135 | | | 0.520 (0.258-0.690) | 0.167 |
|  |  |  |  |  | Yes | 0.128 (-0.113-0.656) |  |  |  | 0.304 (0.162-0.637) |  |
| Frequency of administration | | | biweekly | | | 0.159 (0.124-0.587) | 0.633 | | | 0.365 (0.325-0.565) | 0.639 |
|  |  |  | Once weekly | | | 0.587 (-0.041-0.727) |  |  |  | 0.566 (0.233-0.698) |  |
|  |  |  | Once daily | | | 0.339 (-0.035-0.717) |  |  |  | 0.481 (0.229-0.682) |  |
|  |  |  | Twice daily | | | 0.586 (0.026-0.725) |  |  |  | 0.566 (0.264-0.690) |  |
| Adherence level (CQR-5) | | | Low | | | 0.088 (-0.119-0.622) | **<0.0001*** | | | 0.294 (0.152-0.596) | **<0.0001*** |
|  |  |  | High | | | 0.620 (0.186-0.760) |  |  |  | 0.596 (0.327-0.730) |  |
| Disease activity | | | Low | | | 0.760 (0.586-0.848) | A^a^: **0.005***  B^b^**: <0.0001***  C^c^**: <0.0001*** | | | 0.730 (0.565-0.782) | A^a^: **0.005***  B^b^: **<0.0001***  C^c^: **<0.0001*** |
|  |  |  | Moderate | | | 0.620 (0.125-0.727) |  |  |  | 0.596 (0.321-0.696) |  |
|  |  |  | High | | | 0.069 (-0.166-0.516) |  |  |  | 0.264 (0.140-0.503) |  |
|  | | | **Spearman’s correlation coefficient** | | | |  | | | **Spearman’s correlation coefficient** |  |
| Age | | | -0.114 | | | | 0.067 | | | -0.149 | **0.016*** |
| BMI | | | -0.165 | | | | **0.008*** | | | -0.189 | **0.002*** |
| Disease duration | | | -0.186 | | | | **0.003*** | | | -0.211 | **0.001*** |
| Number of complications | | | -0.265 | | | | **<0.0001*** | | | -0.294 | **<0.0001*** |
| Number of chronic diseases | | | -0.158 | | | | **0.011*** | | | -0.139 | **0.024*** |
| Number of DMARDs | | | 0.055 | | | | 0.378 | | | 0.027 | 0.659 |
| Number of RA medications | | | -0.069 | | | | 0.267 | | | -0.097 | 0.119 |
| Number of total medications | | | -0.165 | | | | **0.008*** | | | -0.177 | **0.004*** |
| Duration of medication intake | | | -0.072 | | | | 0.247 | | | -0.106 | 0.089 |
| ESR | | | -0.298 | | | | **<0.0001*** | | | -0.294 | **<0.0001*** |
| CDAI score | | | -0.502 | | | | **<0.0001*** | | | -0.519 | **<0.0001*** |
| Necessity score | | | -0.185 | | | | **0.003*** | | | -0.169 | **0.006*** |
| Concerns score | | | -0.143 | | | | **0.021*** | | | -0.154 | **0.013*** |

JD: Jordanian Dinar, RA: rheumatoid arthritis, CVD: cardiovascular disease, DMARD: disease-modifying anti-rheumatic drug, NSAIDs: non-steroidal anti-inflammatory drugs, CQR: compliance questionnaire for rheumatology, ESR: erythrocyte sedimentation rate, CDAI: clinical disease activity index, HR-QOL: health-related quality of life, VAS: visual analogue scale, BMI: body mass index

*significant at 0.05 level.

**single: include unmarried, divorced, and widow.

** *High educational level = diploma degree or higher, Low educational level includes illiterate, primary, secondary, and high school.

****low: less than 700 USD, high: 700 USD or more.

a: the significant difference between low and moderate disease activity groups.

b: the significant difference between moderate and high disease activity groups.

c: the significant difference between high and low disease activity groups.
